# Study on strength characteristics and thickening characteristics of classified-fine cemented backfill in gold mine

**DOI:** 10.1038/s41598-023-35254-w

**Published:** 2023-05-24

**Authors:** Xian-qing Wang, Wen Wan, Zhong-liang Yao, Ru-gao Gao, Zhen-xing Lu, Xiao-yu Tang, Bao-jie Fan

**Affiliations:** 1 School of Resource, Environment and Safety Engineering, Hunan University of Science and Technology, Xiangtan, 411201 Hunan China; 2 Feny Co., Ltd., Changsha, 410600 China

**Keywords:** Environmental impact, Civil engineering

## Abstract

For some new mines, the utilization rate of tailings is not satisfactory when using unclassified tailings as backfill aggregate for cemented backfill. At the same time, with the progress of mineral processing technology, the tailings discharged by the concentrator gradually become finer. Therefore, cemented filling with fine-grained tailings as aggregate will become the development direction of filling technology in the future. In this paper, the feasibility of fine particle tailings backfill is studied by taking the particle tailings of-200 mesh as aggregate in Shaling gold mine. The calculation shows that the utilization rate of tailings is increased from 45.1% to 90.3% by using-200 mesh tailings as filling aggregate. The response surface central composite design method (RSM-CCD) was used to study the strength of backfill with alkali-activated cementitious material as binder by taking the mass concentration of backfill slurry and sand-binder ratio as input factors. The results show that the 28-day strength of the backfill with graded fine-grained tailings as filling aggregate can reach 5.41 MPa when the sand-binder ratio is 4, which can fully meet the needs of the mine for the strength of the backfill. The thickening test of-200 mesh fine particle tailings was carried out by static limit concentration test and dynamic thickening test. In the case of adding 35 g/t BASF 6920 non-ionic flocculant, the concentration of 64.74% tail mortar can reach 67.71% after 2 h of static thickening, and the concentration can reach 69.62% after 2 h of static thickening. The feeding speed of thickener should be controlled between 0.4 and 0.59 t/(m^2^ h). In this case, the underflow concentration of thickener is relatively high, which is 64.92–65.78%, and the solid content of overflow water is less than 164 ppm. The conventional full tailings thickening process was improved by using the design of high-efficiency deep cone thickener and vertical sand silo. The feasibility of fine-grained tailings as filling aggregate was demonstrated by combining the filling ratio test of fine-grained tailings, the data of thickening test and the improved thickening process. The research results can provide reference for other mines to use fine-grained tailings as filling aggregate to design filling system.

## Introduction

Tailings are industrial solid wastes generated during the development and utilization of mineral resources, which are mainly stored on the surface in the form of tailings reservoirs^1^. The existence of the tailings reservoir, while occupying a large amount of land resources, the residual chemical agents, free heavy metal ions and pollutants produced after weathering in the tailings will infiltrate into the underground with the water flow, causing pollution to the soil and groundwater resources^2,3^. The dried fine-grained tailings in the tailings reservoir are easy to cause dust pollution when encountering windy weather, which seriously affects the normal life order of the surrounding residents^4,5^. At the same time, with the continuous accumulation of tailings in the tailings reservoir, it is easy to cause geological disasters such as debris flow and flash flood due to the dam break of the tailings reservoir^6,7^. The existence of tailings reservoir poses a potential threat to the surrounding environment and people's life. Filling mining method is to backfill the mined-out area with tailings produced by mineral processing supplemented by cement and water. It can not only reduce the discharge of tailings and control the pressure of mining site, but also prevent surface subsidence and improve the recovery rate of ore^8,9^. Due to the above characteristics, filling mining method has become the preferred mining method for green mine construction^10^. The ratio and concentration of backfill and the selection of filling aggregate determine the strength of backfill^11,12^. For this reason, Some researchers^13^ established a strength prediction model of backfill by using BP neural network and optimized its ratio based on the results of indoor physical test and ratio test of tailings particles^14^. Wen et al.^15^ used unclassified tailings as filling aggregate, and introduced fuzzy comprehensive evaluation system to obtain the optimal ratio of filling slurry. Wu et al.^16^ studied the ratio of whole tailings cementitious materials by orthogonal test and established a regression prediction model of backfill. Based on the response surface analysis method, Fu et al.^17^ studied the influence of filling slurry mass fraction, binder dosage and filling aggregate ratio on the strength of backfill at different ages.

The tailings thickening process is an important part of the cemented filling of the tailings paste^18,19^. The underflow concentration after thickening greatly affects the operating cost of the mine cemented filling. Therefore, Some researchers^20,21^ studied the effect of flocculant unit consumption on the sedimentation rate of tail mortar from the mechanism of flocculant on the sedimentation process of tail mortar. Eswaraiah et al.^22^ studied the effect of different kinds of flocculants on the settlement of tailings mortar under different pH conditions. Wang et al.^23^ studied the effect of flocculant addition on the thickening and sedimentation of tailings with copper-molybdenum tailings mortar as the research object. Some researchers^24,25^ have studied the mechanism of tailings flocculation and established a quantitative relationship between flocculation conditions and the volume characteristics of tailings structure.

The above scholars have studied the recycling of tailings with full tailings or graded coarse-grained tailings as the research object^26,27^, but did not consider the utilization rate of tailings. Using whole tailings or coarse tailings as filling aggregate, there are still some mines with low utilization rate of tailings. At the same time, with the progress of mineral processing technology, the tailings particles discharged by the concentrator will be finer and finer^28^.

At present, most mines use full tailings as filling aggregate to fill the goaf^29,30^. However, because the backfill is a composite material composed of whole tailings, cementitious material and water^31^, the volume of whole tailings produced by one cubic meter of ore is often larger than one cubic meter when it is prepared into filling body. This makes it difficult to achieve the goal of no waste discharge when the mine uses unclassified tailings as filling aggregate. In order to further improve the utilization rate of tailings, this paper combines the actual situation of a gold mine in Shandong, and divides the total tailings into two parts. The coarse-grained tailings above 200 mesh are sold as building materials^32,33^, and the fine-grained tailings below 200 mesh are used as filling body aggregate to fill the goaf. At the same time, with the improvement of beneficiation technology and the improvement of ore resource recovery rate, the tailings particle size produced by the concentrator will gradually decrease. Filling technology with fine particles as filling aggregate will become a hot issue in the future research of filling technology.

Using-200 mesh fine-grained tailings as filling aggregate faces two major technical problems: compared with full tailings or coarse-grained tailings, fine-grained tailings have slower flocculation and sedimentation speed, and the underflow concentration obtained by thickener is lower in the same time. Fine tailings are used as filling aggregate, and traditional cementing agent is used as filling body of cementing material. Its strength is difficult to meet the mining demand.

In view of the above mentioned problems, this paper takes the whole tailings of a gold mine in Shandong Province as the research object, uses the laboratory water screening method to classify the whole tailings, and uses the-200 mesh fine-grained tailings as the test object. Using the similar test, the fine-grained tailings were subjected to the thickener dynamic flocculation sedimentation simulation test. Based on the tailings sedimentation theory, the sedimentation mechanism of the fine-grained tailings flocculation sedimentation process was analyzed. The self-developed cementing agent of Feiyi Co., Ltd. was used to design the ratio test of the filling body with-200 mesh tailings as aggregate by RMS-CCD method. The strength and its influencing factors of the filling body for 3 days, 7 days and 28 days under different cementing agent and tailings ratio and different slurry mass concentration were studied. The feasibility of filling operation with graded fine-grained tailings as filling aggregate in a gold mine in Shandong Province was analyzed. The research results can provide theoretical and experimental reference for the filling design of fine-grained tailings.

## Project overview

**Figure 1:**
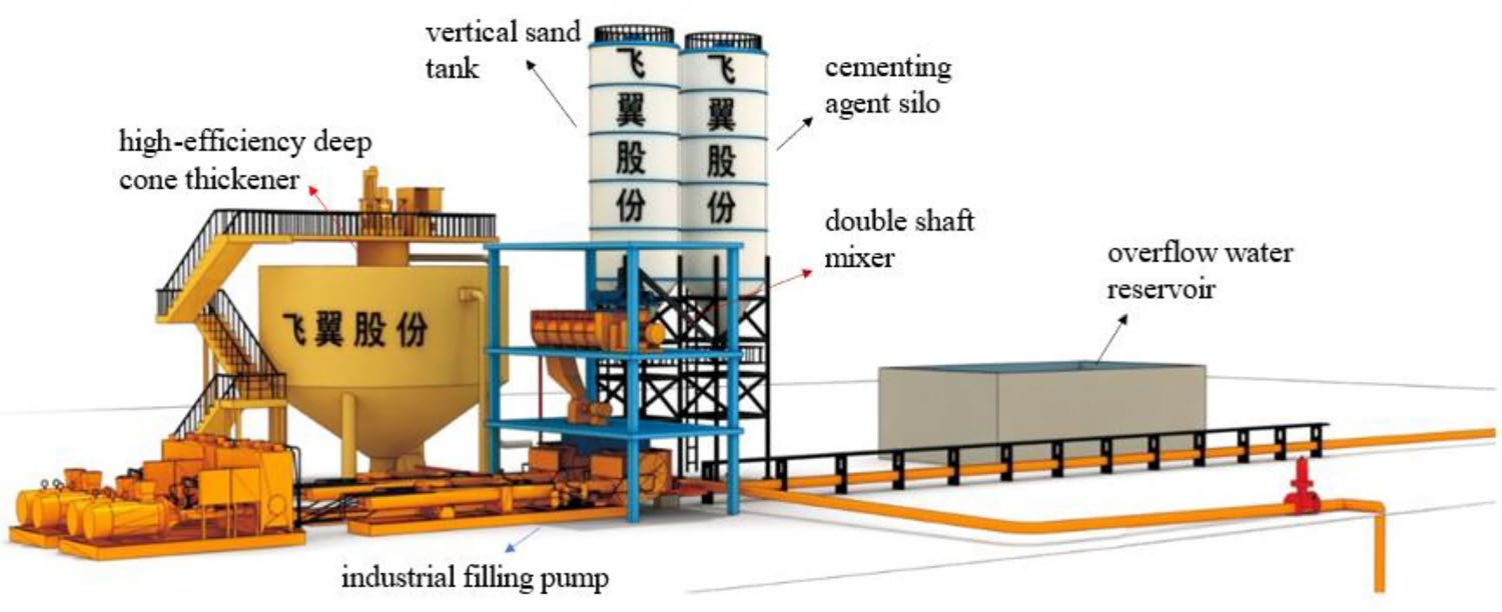
The flow-process diagram of backfill.

The shaling gold mine will be built into a super-large underground gold mine with a mining scale of 12,000 tons/day and an annual output of about 10 tons of gold. The daily empty area volume is about 4300 m^3^, the daily average tailings production is about 11300t, and the proportion of-200 mesh tailings is about 50%. The mine is mined by filling mining method, and the average daily filling amount of tailings is about 5100 tons. If the unclassified tailings are used for filling, the tailings utilization rate is 45.1%. In order to improve the utilization rate of tailings, it is decided to use-200 mesh tailings as filling aggregate for filling. The principle scheme of filling system construction is as follows : after the general tailings of the concentrator are graded by high-frequency vibrating screen, the coarse-grained tailings (tailings on the high-frequency vibrating screen) are further processed and sold as building materials, while the fine-grained tailings (tailings under the high-frequency vibrating screen) enter the slurry pool for filling. The utilization rate of tailings increased from 45.1% to 90.3%. The feasibility of fine-grained tailings as filling aggregate (backfill strength, flocculation sedimentation concentration) is studied. The flow-process is shown in Fig. 1. The diluted fine-grained tailings are transported to the thickener for flocculation and sedimentation, and the thickener underflow is transported to the vertical sand silo for storage and further settlement. The settled tailings mortar and the cementing agent stored in the cementing agent silo are transported to the mixer for full stirring to prepare the filling slurry. The prepared filling slurry is transported by the filling industrial pump to the filling area through the filling pipeline (Table 1).

**Table 1:** Composition and ratio of new type cementing powder.

The raw material name	Blast furnace slag	Bender clinker	Lime	Gypsum	Activating agent	Total
Basic ratio	70–80%	0–5%	3–5%	8–10%	2–3%	100%

## Physical and chemical properties of materials

The tailings used in the test were taken from the Shanling gold mine. The physical properties of the whole tailings are determined by laboratory test, and the results are shown in Table 2. The chemical composition of the whole tailings is shown in Table 3. The cementing powder used in the test is a slag-based cementitious material. The material is prepared by grinding the slag produced during the smelting of pig iron or other metals and adding alkali activator. The composition and ratio of raw materials are shown in Table 1.

**Table 2:** Physical properties of total tailings.

True density (t/m^3^)	Bulk density (t/m^3^)	Percentage of void (%)	pH value
2.68	1.10	59.1	7.5

**Table 3:** Main chemical analysis of total tailings (mass fraction (%)).

SiO_2_	Al_2_O_3_	K_2_O	Fe_2_O_3_	TiO_2_	CaO
50.17	22.70	2.68	2.59	0.946	0.806

**Figure 2:**
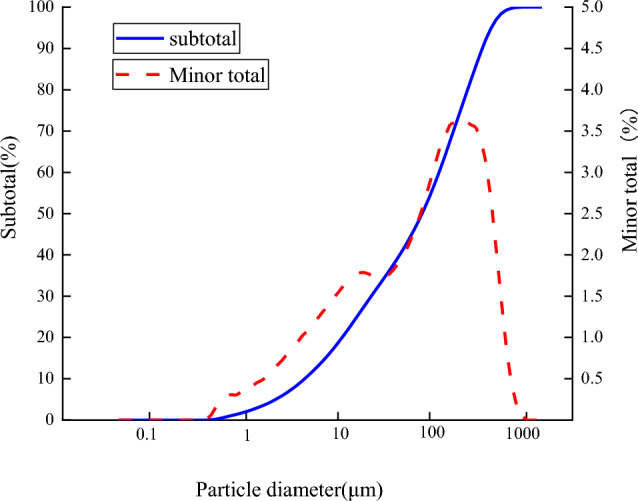
Particle size distribution of unclassified tailings.

**Figure 3:**
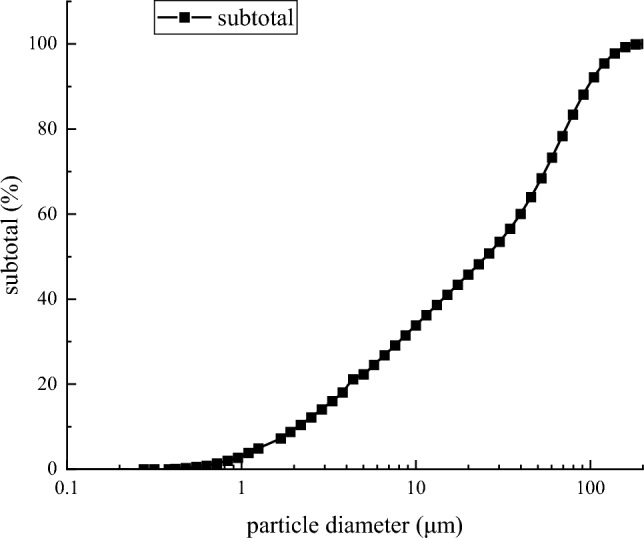
Particle size distribution of -200 mesh tailings.

The particle size composition of the gold mine tailings was analyzed by BT-9300ST laser particle size analyzer, and the results are shown in Fig. 1. It can be seen from Fig. 2 that the-200 mesh tailings particles account for 47.63% of the total tailings. The whole tailings were screened, and the graded tailings below 200 mesh were retained as the backfill aggregate to carry out the backfill ratio test. The particle size distribution of-200 mesh tailings after screening is shown in Fig. 3.

## Backfill strength test

### RSM-CCD experimental design

In this paper, the solid content of the backfill slurry (the mass concentration of the backfill slurry ) and the sand-binder ratio of the backfill slurry were used as input factors, and the unconfined compressive strength of the backfill for 3 days, 7 days and 28 days was used as the response value to study the effect of sand-binder ratio and mass concentration of the backfill slurry and their interaction on the unconfined compressive strength of the backfill. The preliminary exploratory test shows that when the sand-binder ratio of the backfill slurry is 4 ~ 8 and the mass concentration is 70–74%, it can not only meet the demand of the backfill slurry for fluidity, but also obtain a higher backfill strength. Therefore, the mass concentration of backfill slurry and the sand-binder ratio of the backfill slurry was selected as the input factor in the test, and the range was 4 ~ 8 and 70–74% respectively. The CCD method in Design-Experts software was used to design two-factor (sand-binder ratio of backfill slurry, mass concentration of backfill slurry) three-level (− 1,0,1) test^34^. The experimental factors and levels are shown in Table 4.

**Table 4:** Factors and levels of experiment of Response Surface Analysis.

Test factors	Factor level		
	− 1	0	1
A the sand-binder ratio of the backfill slurry	4	6	8
B the solid content of the backfill slurry (%)	70	72	74

### Recipe of backfill slurry

According to the test factors and levels designed in Table 4, the recipe of backfill slurry was carried out. The test results are shown in Table 5.

**Table 5:** Design and results of Response Rurface Analisis.

Test number	Factor coding	Factor 1	Factor 2	Test result			
	X_1_	X_2_	Sand-binder ratio	Mass concentration (%)	3 days compressive strength (MPa)	7 days compressive strength (MPa)	8 days compressive strength (MPa)
1	1	1	8	74	0.785	0.987	1.520
2	0	− 1	6	70	0.784	1.336	1.970
3	0	0	6	72	0.884	1.701	2.110
4	0	1	6	74	1.306	2.297	2.812
5	− 1	0	4	72	2.401	3.481	4.317
6	1	− 1	8	70	0.492	0.625	0.765
7	− 1	− 1	4	70	1.709	2.881	3.467
8	1	0	8	72	0.555	0.891	0.938
9	− 1	1	4	74	3.511	4.668	5.410

Taking the test factor A and the test factor B as the independent variables, and the compressive strength of the filling body Y as the response, the nonlinear regression analysis of the test data in Table 5 can be used to classify the compressive strength response function of the backfill with fine particle tailings as aggregate for 3 days, 7 days and 28 days.

3-day compressive strength response function:

1 $$Y_{3} = 4.556A - 4.658B - 0.094A \cdot B + 0.146A^{2} + 0.038B^{2} + 148.51$$

7-day compressive strength response function:

2 $$Y_{7} = 4.269A - 3.094B - 0.089A \cdot B + 0.119A^{2} + 0.027B^{2} + 93.1$$

28-day compressive strength response function:

3 $$Y_{28} = 3.199A - 6.544B - 0.074A \cdot B + 0.11A^{2} + 0.051B^{2} + 220.005$$

In the formula: A is the sand-binder ratio of the backfill slurry, B is $\cdot$ the solid content of the backfill slurry (%).

## Thickening test

According to the flocculant selection test carried out by the filling laboratory of Feiyi Co., Ltd., when the concentration of fine-grained tailings slurry is 9.52%, the flocculant is BASF 6920 non-ionic flocculant, and the flocculant addition amount is 35 g/t, the tailings sedimentation rate is relatively fast, and the solid content of the clear liquid above the water–sand interface is the lowest. The static limit concentration test and dynamic thickening similarity test were carried out on the tailings slurry with a concentration of 9.52%. The concentration of the tailings slurry discharged by the concentrator of the gold mine is 30%. The static limit concentration test of the tailings slurry with a concentration of 30% is carried out. The test results can provide a test reference for the design of the tailings thickening system in the filling station.

### Static limit concentration test

**Figure 4:**
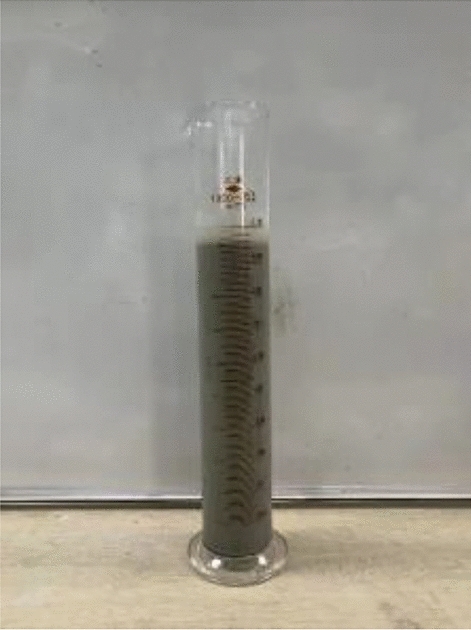
Static limit concentration test.

The fine-grained tailings slurry with 9.52% and 30% concentrations was prepared by using a 1000 mL measuring cylinder, in which BASF 6920 nonionic flocculant was added, and the flocculant addition amount was 35 g/t, as shown in Fig. 4. Observe the sedimentation effect of tailings and record the sedimentation data according to time. The experimental results are shown in Tables 6 and 7.

**Table 6:** Results of static settlement limit of 9.52% concentration slurry.

Sedimentation time (s)	Height of boundary between overflow water and tail mortar (mL)	Tailing slurry concentration (%)
10	750	12.45
20	530	17.07
30	330	25.75
40	260	31.33
50	235	33.96
60	220	35.76
120	175	42.52
180	155	46.42
240	150	47.51
300	145	48.65

**Table 7:** Results of static settlement limit of 30% concentration slurry.

Sedimentation time (h)	Height of boundary between over-flow water and tail mortar (mL)	Tailing slurry concentration (%)
2	410	57.66
3	380	60.49
4	368	61.71
5	355	63.08
6	340	64.74
7	327	66.25
8	315	67.71
10	300	69.62
12	290	70.96
16	280	72.35
24	278	72.64

### Dynamic thickening test

1. Preparation of flocculant
  The flocculant is BASF 6920 nonionic flocculant, which is prepared into 35 g / t diluent at room temperature for use. The preparation process is shown in Fig. 5.

**Figure 5:**
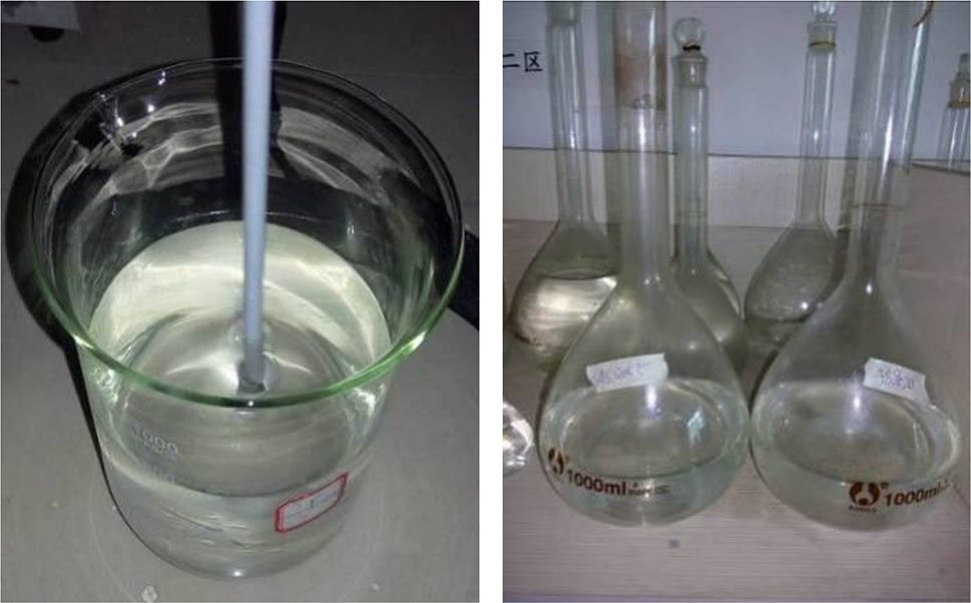
Flocculant preparation process.
2. Dynamic thickening test
  Four peristaltic pumps were used in the dynamic test to pump diluted water, flocculant and fine-grained tailings into the feeding system of the thickening test device, and the underflow ore sample was pumped out from the bottom of the test device. Diluted water using tap water; the flocculant was added through two different administration points, and the amount of addition was based on the data obtained from the static test. The mass concentration of the tailings is about 9.52%, and then placed in a 100 L barrel with an electric mixer to fully stir evenly, and finally pumped into the pipeline. By calculating and adjusting the speed of the peristaltic pump, the flocculant and tailings samples can reach the optimal addition ratio of the static test and simulate the thickening test results under different conditions. When the mud layer height is 175 mm, the overflow water is sampled and measured, and when the mud layer height is 350 mm, the underflow concentration is sampled and measured.
  The dynamic thickening test uses a 100 mm diameter thickener simulation test device, as shown in Fig. 6.

**Figure 6:**
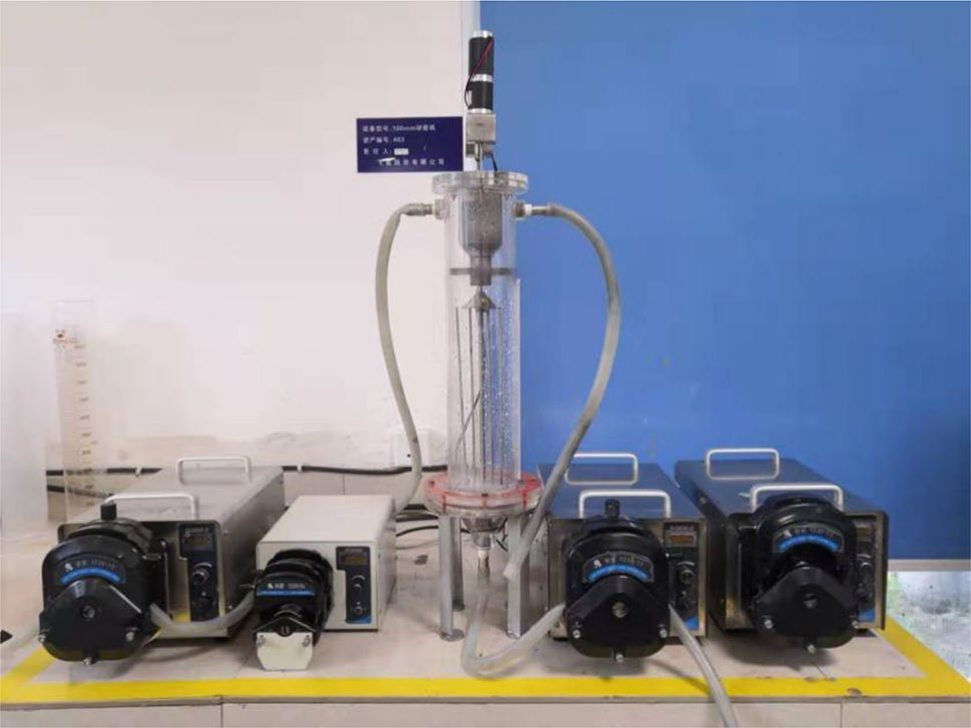
100 mm diameter thickener simulation test equipment.
3. Test result
  The dynamic thickening sedimentation test mainly studies the influence of different feeding speeds on the clarity of overflow water and the concentration of underflow at a feed concentration of about 9.52%. The test results are shown in Table 8 and Fig. 7.

**Table 8:** tailings dynamic thickening test results.

Test serial number	Delivery rate (t/m^2^ h)	Feeding concentration (%)	Dose of flocculant (g/t)	Underflow concentration (%)	Underflow bulk density(t/m^3^)	Overflow water solid content (ppm)	Underflow yield stress (Pa)
1	0.40	9.52	35	65.78	1.72	94.5	186.471
2	0.49	9.52	35	65.56	1.72	154.2	170.900
3	0.59	9.52	35	64.92	1.71	163.2	144.280
4	0.81	9.52	35	63.02	1.68	198.8	56.673
5	1.02	9.52	35	61.96	1.66	242.9	27.150

**Figure 7:**
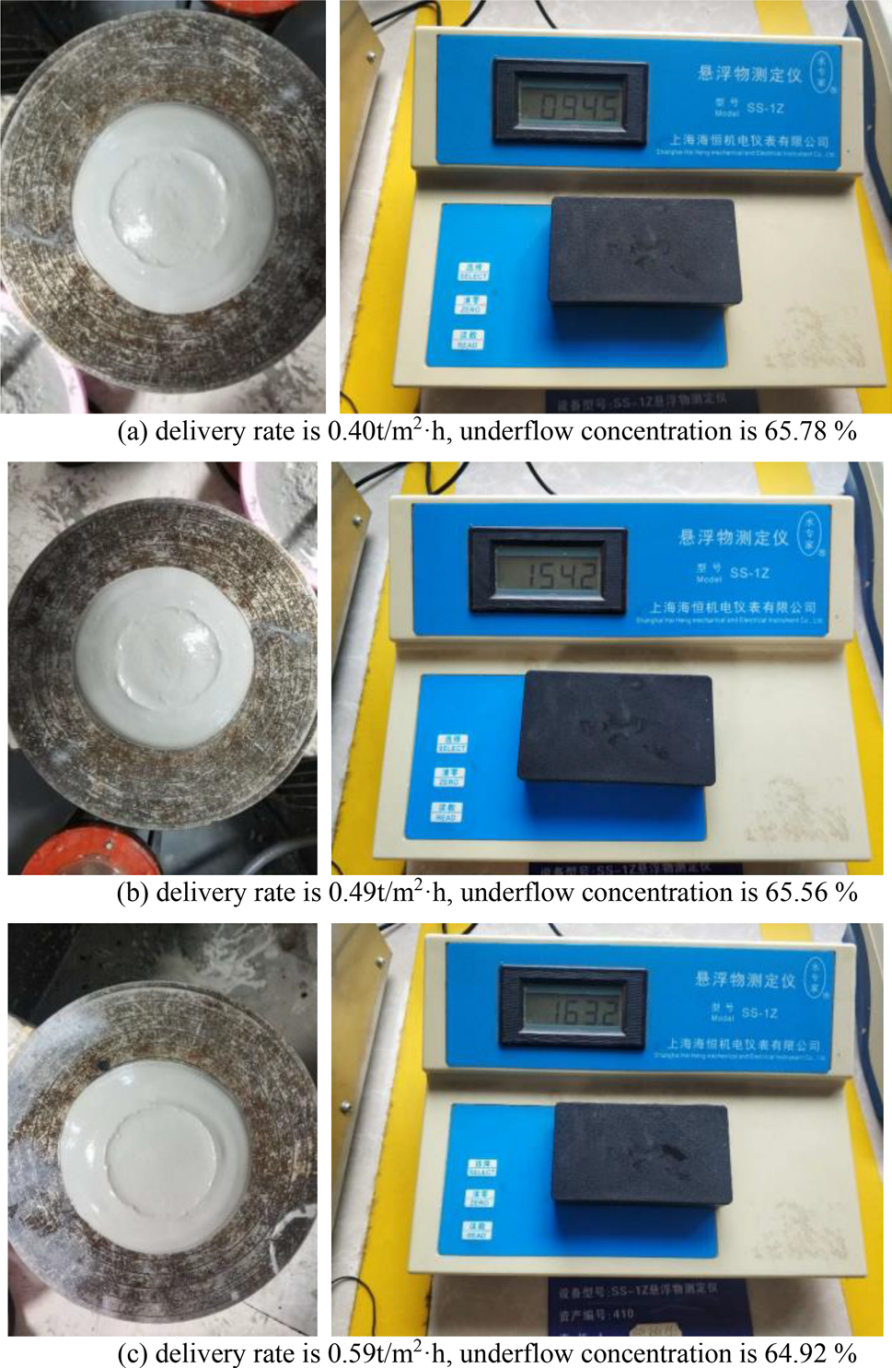
Dynamic dense sedimentation test diagram.

## Analyzed and discussed

### Analysis and discussion of recipe of backfill slurry results

#### Reliability analysis and error estimation of response surface fitting function

According to engineering experience, the cost of cementing agent accounts for more than 70% of the filling operation cost. Therefore, based on the needs of different mines, adjusting the slurry concentration and sand-binder ratio in the range of 70% ~ 74% and 4 ~ 8 can greatly reduce the filling operation cost of the mine. The reliable empirical formula of backfill strength can provide reference for the adjustment of filling slurry concentration and cement-sand ratio in accordance with the requirements of working conditions. Now the hypothesis test-P test is carried out on the fitting function model with the compressive strength of the backfill as the response. The variance of each explanatory variable in the response surface regression model is shown in Table 9. In the response surface fitting function model, the significance level α = 0.05, if the P value of an explanatory variable in the fitting function is less than 0.05, it indicates that the item is a significant item in the model. If the P value of the item is greater than 0.1, it indicates that the item is a non-significant item in the model. When fitting the model function, this item should be omitted to simplify the model function. From Table 9, it can be seen that the P values of the explanatory variable B^2^ item in each model are all greater than 0.1, so the explanatory variable B^2^ item is a non-significant item in the model function. The model function is modified, and the modified unconfined compressive strength response function of backfill is as follows:

**Table 9:** Analysis of variance with regression model of different response surfaces.

	Source	Quadratic sum	Degree of freedom	Mean square	F value	*P* value
3-day compressive strength	model	8.02	5	1.60	49.43	0.0044
	A	5.59	1	5.59	172.04	0.0010
	B	1.14	1	1.14	35.16	0.0096
	AB	0.5693	1	0.5693	17.53	0.0248
	A^2^	0.6825	1	0.6825	21.02	0.0195
	B^2^	0.0457	1	0.0457	1.41	0.3208
7-day compressive strength	model	14.72	5	2.94	169.47	0.0007
	A	12.12	1	12.12	697.73	0.0001
	B	1.61	1	1.61	92.81	0.0024
	AB	0.5077	1	0.5077	29.23	0.0124
	A^2^	0.4560	1	0.4560	26.26	0.0144
	B^2^	0.0233	1	0.0233	1.34	0.3304
28-day compressive strength	model	19.4	5	4.85	110.18	0.0002
	A	16.57	1	16.57	376.50	< 0.0001
	B	2.09	1	2.09	47.46	0.0023
	AB	0.3528	1	0.3528	8.02	0.0473
	A^2^	0.3851	1	0.3851	8.75	0.0416
	B^2^	0.0819	1	0.0819	2.61	0.2047

3-day compressive strength response function:

4 $$Y_{3} = 4.556A{ + }0.784B - 0.094A \cdot B + 0.146A^{2} - 47.302$$

7-day compressive strength response function:

5 $$Y_{7} = 4.269A{ + }0.794B - 0.089A \cdot B + 0.119A^{2} - 46.769$$

28-day compressive strength response function:

6 $$Y_{28} = 3.199A{ + }0.741B - 0.074A \cdot B + 0.11A^{2} - 42.083$$

In the formula: A is the sand-binder ratio of the backfill slurry, B is the solid content of the backfill slurry (%).

The adjusted coefficient of determination (Adjusted R^2^) of the fitting function of the compressive strength of the backfill at 3 days, 7 days and 28 days after correction is 0.9648,0.9898 and 0.982, respectively. In general, the fluctuation of the value is small and close to 1, indicating that the fitting function has high reliability.

**Figure 8:**
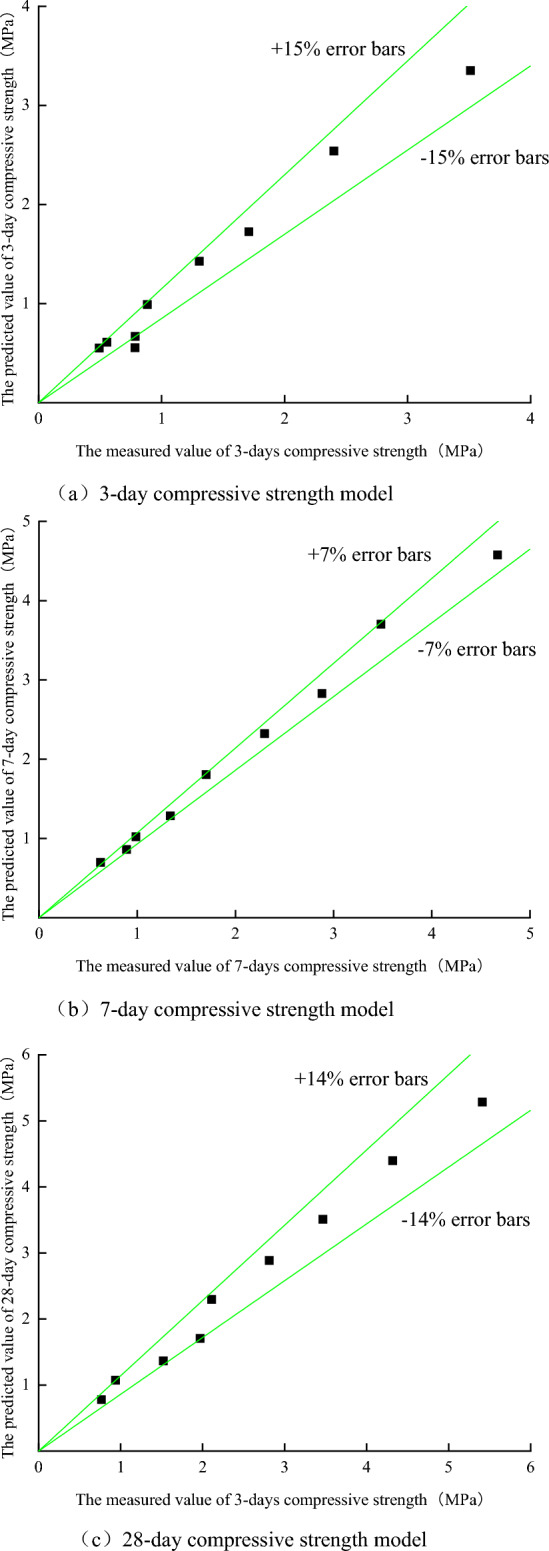
Error curve between actual value and calculated value of response surface model.

Taking the measured value and predicted value of the strength of the backfill as the ordinate and abscissa values of the points in the error analysis diagram, the error analysis of the compressive strength prediction model of the backfill is carried out, as shown in Fig. 8. It can be seen from Fig. 8 that the error between the predicted value and the actual value calculated by the 3-day compressive strength prediction model is less than 15% except for one point, the error of the 7-day compressive strength prediction model is less than 7%, and the error of the 28-day compressive strength prediction model is less than 14%. It shows that the prediction error of the unconfined compressive strength prediction model based on the response surface analysis method is within the controllable range. The model can be used as an empirical formula for the compressive strength of the filling body, and it can be used as a reference for the mine to adjust the concentration and ratio of the filling slurry according to the needs of different working conditions.

#### Response surface single factor sensitivity analysis

The independent variable of the response function of the compressive strength value of the backfill is rewritten as the factor coding form:

3-day compressive strength response function:

7 $$Y_{3} = - 0.965X_{1} + 0.4362X_{2} - 0.377X_{1} \cdot X_{2} + 0.584X_{1}^{2} + 0.991$$

7-day compressive strength response function:

8 $$Y_{7} = - 1.42X_{1} + 0.518X_{2} - 0.356X_{1} \cdot X_{2} + 0.4775X_{1}^{2} + 1.78$$

28-day compressive strength response function:

9 $$Y_{28} = - 1.66X_{1} + 0.59X_{2} - 0.297X_{1} \cdot X_{2} + 0.439X_{1}^{2} + 2.3$$

**Figure 9:**
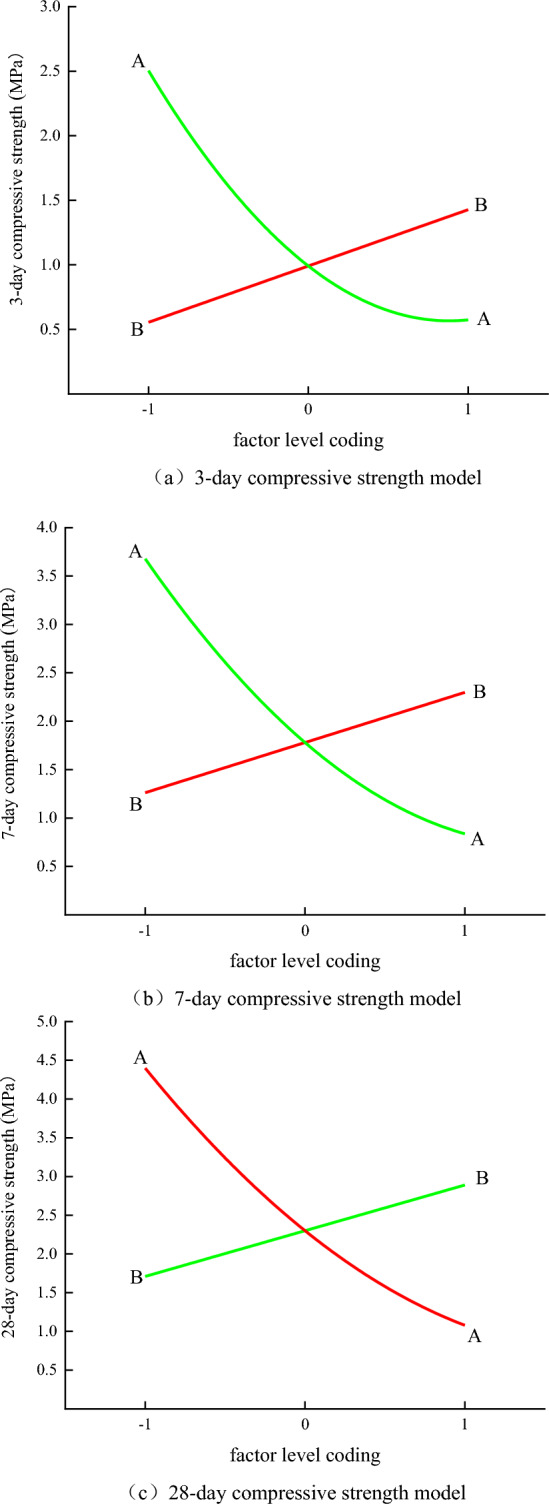
Factor level disturbance diagram.

In the formula, Y_3_, Y_7_ and Y_28_ are the compressive strength of the filling body for 3 days, 7 days and 28 days respectively.Among them, X_1_ is the horizontal coding of total tailings mass concentration factor, and the value range is-1 ~ 1.X_2_ is the horizontal coding of flocculant unit consumption factor, and the value range is-1 ~ 1.Let the value of factor level code X_1_ and X_2_ be zero respectively, and make the disturbance diagram of compressive strength response function of 3 days, 7 days and 28 days, as shown in Fig. 9. The magnitude or curvature of the absolute value of the function slope in the response surface function perturbation plot reflects the sensitivity of the response function value to the coding of this factor. The larger the slope absolute value or curvature of the response function is, the more sensitive the response function value is to this factor.

From Fig. 9, it can be seen that factor A (sand-binder ratio of backfill slurry) is a quadratic curve in the disturbance map, and factor B (mass concentration of backfill slurry) is a straight line in the disturbance map. Obviously, the curvature of factor A curve is greater than that of factor B curve, so the sensitivity of compressive strength of backfill to sand-binder ratio is greater than that of backfill slurry mass concentration. On the other hand, from Table 9, it can be seen that the F value of the explanatory variable A in the compressive strength function of the paste at 3 days, 7 days and 28 days is greater than the other explanatory variables, indicating that the sand-binder ratio has the most obvious influence on the compressive strength value, which is consistent with the results of sensitivity analysis using the disturbance map.

#### Comprehensive analysis of compressive strength of backfill

**Figure 10:**
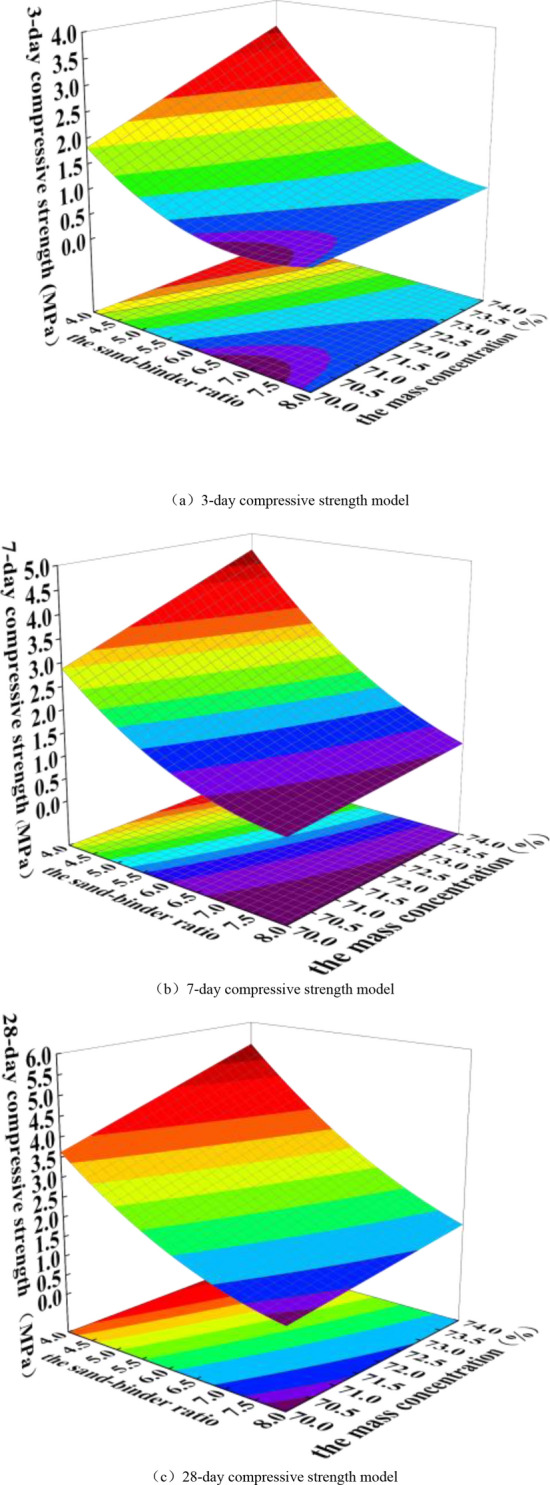
Characteristic analysis diagram of response surface.

The response surface of 3 days, 7 days and 28 days compressive strength was made by using the established prediction model, as shown in Fig. 10. It can be seen from the figure that the 3-day compressive strength of the backfill decreases with the increase of the sand-binder ratio of the backfill slurry, but with the increase of the sand-binder ratio, the 3-day strength of the backfill decreases gradually. When the mass concentration of backfill slurry is 70% and the sand-binder ratio of backfill slurry increases from 4 to 6, the 3-day compressive strength of backfill decreases by 54%. When the sand-binder ratio of backfill slurry increases from 6 to 8, the 3-day compressive strength of backfill decreases by 37%. This shows that the 3-day compressive strength of the backfill increases with the increase of mass concentration and decreases with the increase of sand-binder ratio. This is because the main reason for the formation of the strength of the backfill is that the aggregate in the backfill is bonded to a whole by the product of the hydration reaction of the cementing agent-hydrated silicic acid and hydrated calcium aluminate. To a certain extent, the more the silica-alumina gel phase produced by the hydration of the cementing agent, the greater the cohesion inside the aggregate of the backfill, and the higher the unconfined compressive strength of the backfill^35^. Therefore, the binder is the main factor affecting the strength of the backfill. The higher the mass concentration of the backfill slurry, the higher the aggregate content of the backfill slurry, the higher the aggregate content of the backfill slurry, the easier it is to form a dense skeleton structure, and a good skeleton structure can make the backfill obtain higher bearing capacity.

When the curing period is 3 days, the strength attenuation ratio of the backfill is 54% when the sand-binder ratio increases from 4 to 6, and the strength attenuation ratio of the backfill is 37% as the sand-binder ratio increases from 6 to 8. This is due to the fact that in the early stage of hydration reaction, although the binder content of the latter is less than that of the former, the contact area between the latter and water is larger, so the hydration reaction speed of the latter is faster and more cement is produced, so the strength reduction of the latter is lower than that of the former. When the curing period is 28 days, the hydration in the backfill is more sufficient. At this time, the content of binder agent in the backfill determines the amount of cement generated in the hydration reaction. Therefore, the strength attenuation ratio of the backfill with the sand-binder ratio increasing from 4 to 6 is less than the strength attenuation ratio of the backfill with the sand-binder ratio increasing from 6 to 8.

### Analysis and discussion of thickening test results

#### Analysis of tailings thickening mechanism

**Figure 11:**
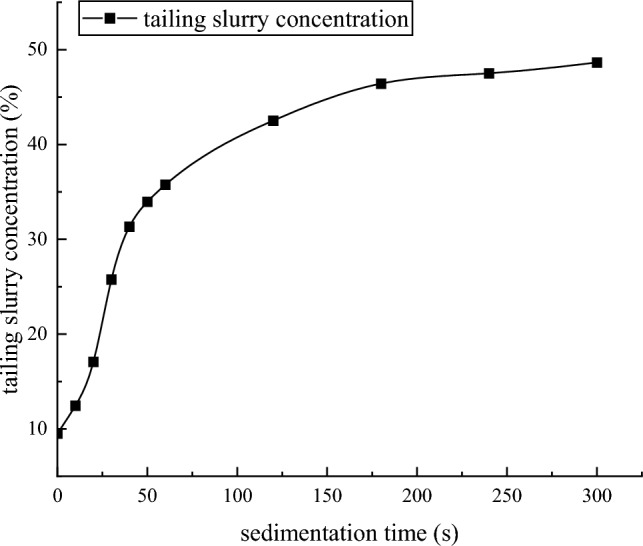
9.52% concentration slurry static sedimentation limit concentration curve.

**Figure 12:**
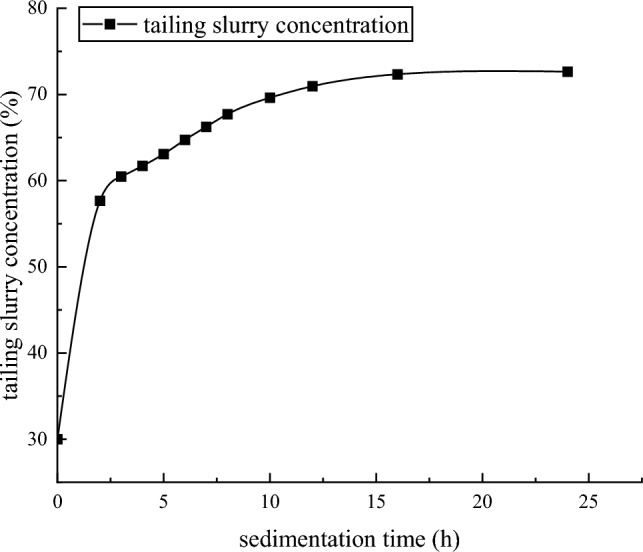
The static sedimentation limit concentration curve of 30% concentration slurry.

The static thickening test is to test the free settlement of tailings particles under the action of gravity, which simulates the process of free settlement of tailings slurry in vertical sand silo. It can be seen from Figs. 11 and 12 that the settlement rate of the interface between overflow water and tailings mortar is related to the concentration of tailings mortar. The lower the concentration of the tail mortar, the faster the sinking speed of the interface. With the increase of the concentration of the tail mortar, the sinking speed of the interface gradually slows down. When the concentration of the tail mortar reaches the limit concentration of static settlement, the sinking speed of the interface decreases to 0.

**Figure 13:**
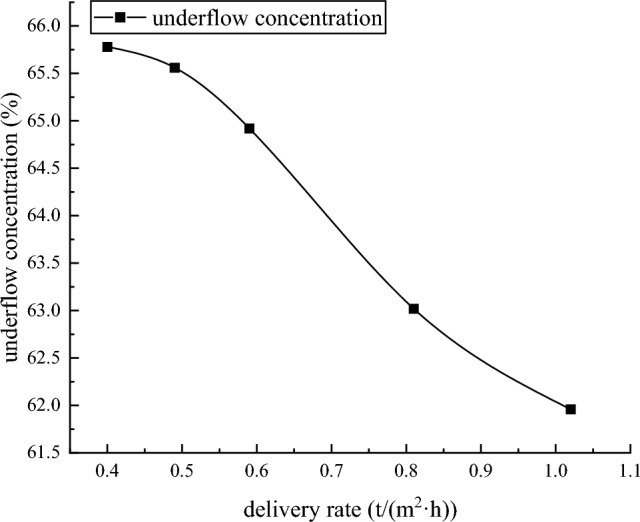
Trend chart of underflow concentration.

**Figure 14:**
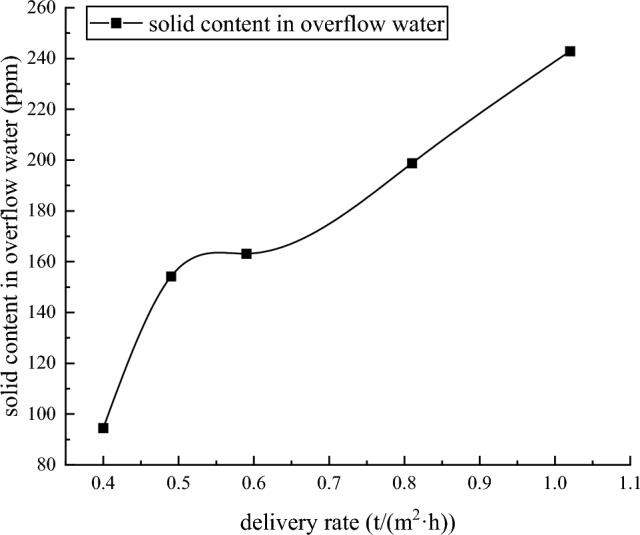
Trend chart of solid content in overflow water.

From Figs. 13 and 14, it can be seen that when the feeding speed of the thickener changes from 0.40 (t/m^2^·h) to 1.25 (t/m^2^·h), the solid content of the overflow water increases from 94.5 ppm to 242.9 ppm, and the underflow concentration decreases from 65.78% to 61.96%. The solid content of overflow water increases with the increase of feeding speed of thickener, and the underflow concentration decreases with the increase of feeding speed of testing machine. This is because the tailings particles will cause a liquid rise slightly smaller than the volume of the tailings particles during the sinking process in the thickener. The increase of the feeding speed of the thickener increases the tailings particles in the same liquid level of the thickener. When the tailings particles in the liquid level are settled, a larger volume of liquid level will rise, while the cross section of the thickener remains unchanged. Therefore, the increase of the feeding speed of the thickener increases the rising speed of the liquid level in the thickener. The increase of the rising speed of the liquid level increases the resistance of the tailings particles during the sinking process. This leads to a longer time for the tailings to complete the flocculation sedimentation. The internal slurry of the thickener is in a dynamic equilibrium state, and the time required for the tailings particles to complete the flocculation and sedimentation process increases, resulting in the failure of sufficient flocculation and sedimentation of some tailings in the thickener. Therefore, when the feeding speed of thickener increases, the underflow concentration decreases and the solid content of overflow water increases.

**Figure 15:**
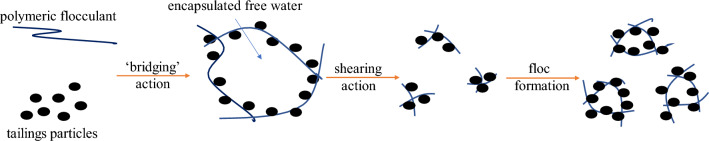
Dynamic flocculation sedimentation process of tailings.

It can be seen from Table 6 that the static maximum sedimentation concentration of the tailings slurry with a concentration of 9.52% was 48.65% by adding 35 g / t BASF 6920 nonionic flocculant. In the dynamic thickening experiment, the underflow concentration of the tailings slurry can reach more than 61.9% after the thickener. This is because the flocculant molecules adsorb the tailings particles on whole tailings, the flocculant molecules adsorb the tailings particles on their chain network structure when they collide with the tailings particles. Under the action of gravity, the tailings particles encapsulate a part of free water to form a relatively large unstable floc structure through the ' bridging ' of flocculant molecules. The formation of floc structure accelerates the sedimentation rate of tailings particles in liquid. When the floc structure sank to the action area of the thickener rake, under the shear stress of the rake, the unstable floc structure chain network with relatively large volume broke and released some free water wrapped inside the floc structure. After part of the wrapped free water is discharged, the broken chain network junction forms a smaller and more stable floc structure during the collision process^36^. The process is shown in Fig. 15. Compared with the static flocculation sedimentation process, the dynamic flocculation sedimentation process of tailings under the action of shear stress not only discharges the free water between the flocs and the flocs, but also releases some free water wrapped in the relatively unstable floc structure formed only under the action of gravity, which further increases the underflow concentration.

#### Optimization design of thickening system

**Figure 16:**
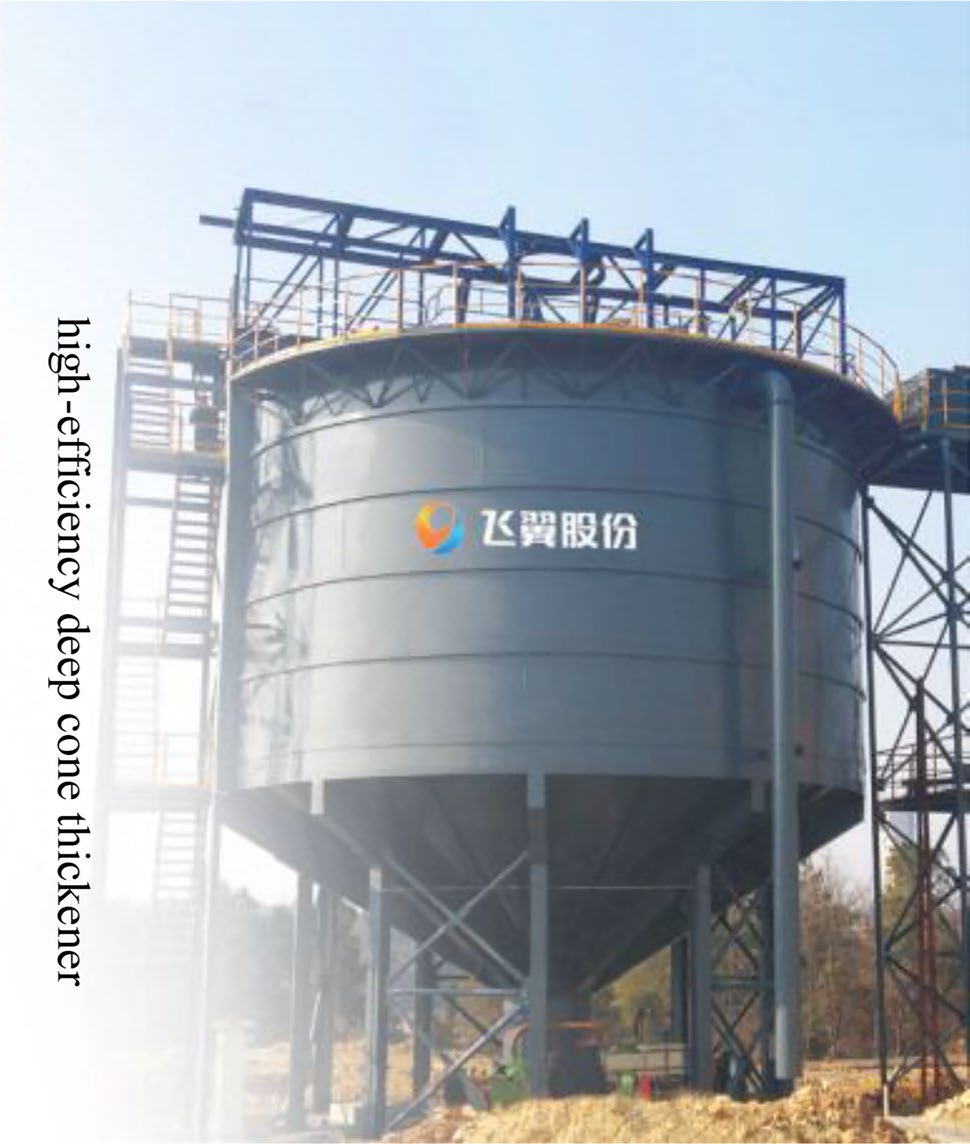
Conventional unclassified tailings thickening process.

**Figure 17:**
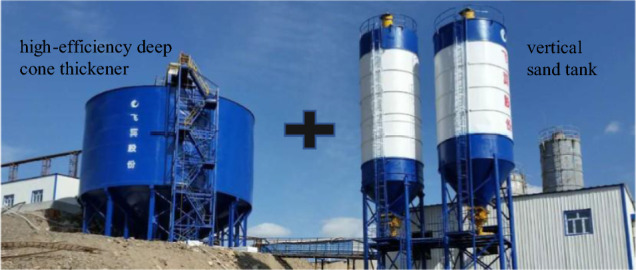
Optimized thickening process.

According to the test results of dynamic thickening, the recommended feeding speed of thickener is 0.4 ~ 0.59 t/(m^2^·h). At this speed, the underflow concentration of thickener is 64.92% ~ 65.78%, and the solid content of overflow water is less than 164 ppm. The underflow concentration of thickener is relatively high and the solid content of overflow water is relatively low. The underflow concentration of the thickener is maintained at about 65%. If the conventional unclassified tailings filling process (as shown in Fig. 16) is followed, the underflow of the high-efficiency deep cone thickener directly enters the mixer and is prepared with the binder to prepare the backfill slurry. Even if the sand-binder ratio is mixed at 4:1, the concentration of the resulting backfill slurry is only about 70%. This leads to a higher strength of the backfill only by adding more binder, which greatly increases the filling operation cost. In order to obtain a higher underflow concentration, so as to achieve the purpose of reducing the filling operation cost, the conventional tailings thickening process only by the high-efficiency deep cone thickener is improved to the high-efficiency deep cone thickener supplemented by the vertical sand silo, as shown in Fig. 17. The improved thickening process is as follows: after the tail mortar is thickened by an efficient deep cone thickener, the underflow of the thickener is transported to a vertical sand silo by a slurry pump for storage and further settlement to obtain a higher concentration of tail mortar.

**Figure 18:**
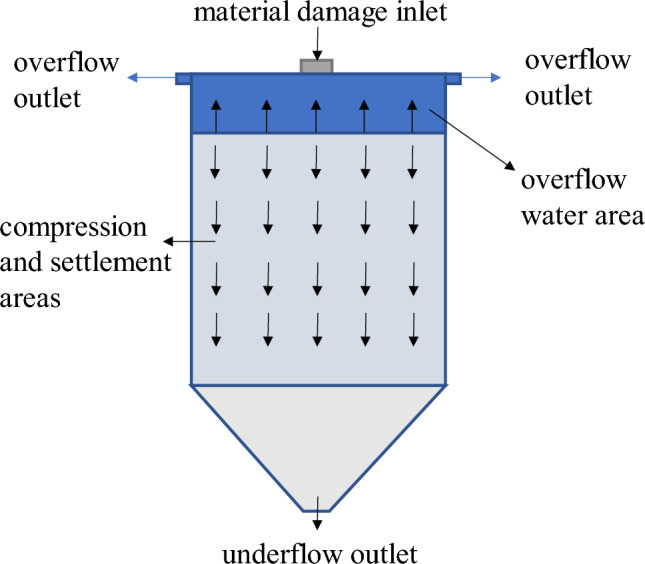
Thickening process of vertical sand silo.

The thickening process in the vertical sand silo is shown in Fig. 18. The thickening process in the vertical sand silo is analyzed. When the feed amount of the vertical sand silo is equal to the discharge amount, the vertical sand silo is in a dynamic equilibrium state. At this time, the concentration of the tail mortar in the sand silo no longer changes with time. At this time, the material balance equation in the vertical sand silo is:

Total material balance:

10 $$Q_{F} = Q_{U} + Q_{0}$$

Solid particle balance in tail mortar:

11 $$Q_{F} \varphi_{F} = Q_{U} \varphi_{U} + Q_{0} \varphi_{0}$$

Liquid balance in tail mortar:

12 $$Q_{F} (1 - \varphi_{F} ) = Q_{U} (1 - \varphi_{U} ) + Q_{0} (1 - \varphi_{0} )$$

Assuming that the overflow water does not contain tailings particles, there are:

13 $$Q_{F} \varphi_{F} = Q_{U} \varphi_{U}$$

14 $$Q_{F} (1 - \varphi_{F} ) = Q_{U} (1 - \varphi_{U} ) + Q_{0}$$

In the formula : *Q*_*F*_, *Q*_*U*_, *Q*_*0*_ are the flux of the vertical sand silo feed port, underflow outlet, and overflow port,, m^3^/h; *φ*_*F*_*、φ*_*F*_*、φ*_*0*_ are the mass fraction of solid particles in the feed inlet, underflow outlet and overflow respectively.

The reasonable design of the vertical sand silo cooperates with the scientific arrangement of the filling time, and the material inside the vertical sand silo is in a dynamic equilibrium. According to Formula (13), the solid flux of the vertical sand silo feed port is about equal to the solid flux of its underflow outlet. According to the static thickening limit concentration test results of the tail mortar with a concentration of 30%, it can be seen that the tail mortar entering the vertical sand bin after the first thickening of the thickener is carried out in the vertical sand bin for more than 2 h. The underflow concentration of the vertical sand bin will reach about 68%. If the time of secondary thickening exceeds 4 h, the underflow concentration can reach about 70%. This greatly reduces the operating costs of mine filling.

At the same time, according to the practical experience, the thickening process only includes the high-efficiency deep cone thickener. Due to the influence of the height of the thickener side wall, the low flow concentration of the thickener will slowly decrease with the increase of the filling working time. The new thickening process composed of a high-efficiency deep cone thickener and a vertical sand bin can not only maintain the stability of the underflow concentration after thickening during the filling operation, but also the new vertical sand bin and thickener can be regarded as a small accident pool. When the backfill system fails and needs to be repaired, if the accident treatment time is not long, the tail mortar can be directly sent to the vertical sand bin and thickener for storage. This design reduces the maintenance cost of the backfill station under the premise of ensuring the mine backfill quality.

## Conclusion

1. The unclassified tailings of Shandong Shaling Gold Mine were graded, and the graded fine-grained tailings below 200 mesh were used as the aggregate of backfill for strength test. The test results show that when the sand-binder ratio is 4 and the mass concentration of the backfill is 74%, the strength of the backfill for 3 days, 7 days and 28 days is 3.511 MPa, 4.668 MPa and 5.41 MPa respectively, which can fully meet the needs of the mine for the strength of the backfill. The calculation results show that compared with the unclassified tailings as the backfill aggregate, the utilization rate of the tailings in the gold mine can be increased from 45.1% to 90.3% when the graded fine-grained tailings below 200 mesh are used as the backfill aggregate.
2. Based on RSM-CCD method, the prediction model of 3 days, 7 days and 28 days compressive strength of backfill was established. The continuity of the model in the test range was tested by the Adjusted R^2^ value of the model. The analysis of the applicability and error of the model showed that the model could be used to estimate the compressive strength of backfill in the range of sand-binder ratio 4 ~ 8 and backfill slurry concentration 70–74%, and the error was less than 15%. The sensitivity of the influence of sand-binder ratio and mass concentration of backfill slurry on the compressive strength of backfill was analyzed by disturbance diagram. The results showed that the sensitivity of backfill strength to sand-binder ratio of backfill slurry was higher than that of mass concentration.
3. In the case of adding 35 g/t BASF 6920 non-ionic flocculant, the static limit concentration test of tailings shows that the concentration of 64.74% tailings mortar can reach 67.71% after 2 h of static thickening, and the concentration can reach 69.62% after 4 h of static thickening. The results of dynamic thickening test show that the feeding speed of thickener should be controlled between 0.4 and 0.59 t/(m^2^·h). At this time, the underflow concentration of thickener is relatively high, which is 64.92% ~ 65.78%, and the solid content of overflow water is less than 164 ppm.
4. The conventional full tailings thickening process was upgraded by using the design of thickener plus vertical sand silo. The upgraded tailings thickening process can ensure the stable supply of underflow concentration. When the secondary thickening time of tailings mortar is more than 2 h, the underflow concentration can reach about 68%. When the thickening time is more than 4 h, the underflow concentration can reach about 70%.
5. Through the recipe of backfill slurry test and thickening test of the backfill in the laboratory and the optimization and upgrading of the on-site thickening system, the feasibility of using fine-grained tailings as backfill aggregate is demonstrated. The test results and optimization design results can provide reference for other mines to use fine-grained tailings as backfill aggregate for backfill system design.

## Data Availability

All data generated or analysed during this study are included in this published article.
